# Supplementary material for: The Role of ZNF143 in Breast Cancer Cell Survival Through the NAD(P)H Quinone Dehydrogenase 1–p53–Beclin1 Axis Under Metabolic Stress
Source: Cells. 2019 Mar 30;8(4):296. doi: 10.3390/cells8040296 (PMC6523662; doi:10.3390/cells8040296)
Supplement: Supplementary file 1 [file cells-08-00296-s001.pdf]

cells

MDPI

Article

A role of ZNF143 on cell survival through the NAD(P)H quinone dehydrogenase 1-p53-Beclin1 axis under metabolic stress in breast cancer cells

A Rome Paek1, Ji Young Mun2, Mun Jeong Jo1, Hyosun Choi3, Yun Jeong Lee3, Heesun Cheong4,5, Jae Kyung Myung4, Dong Wan Hong6, Jongkeun Park6, Kyung-Hee Kim7 and Hye Jin You1,4,*

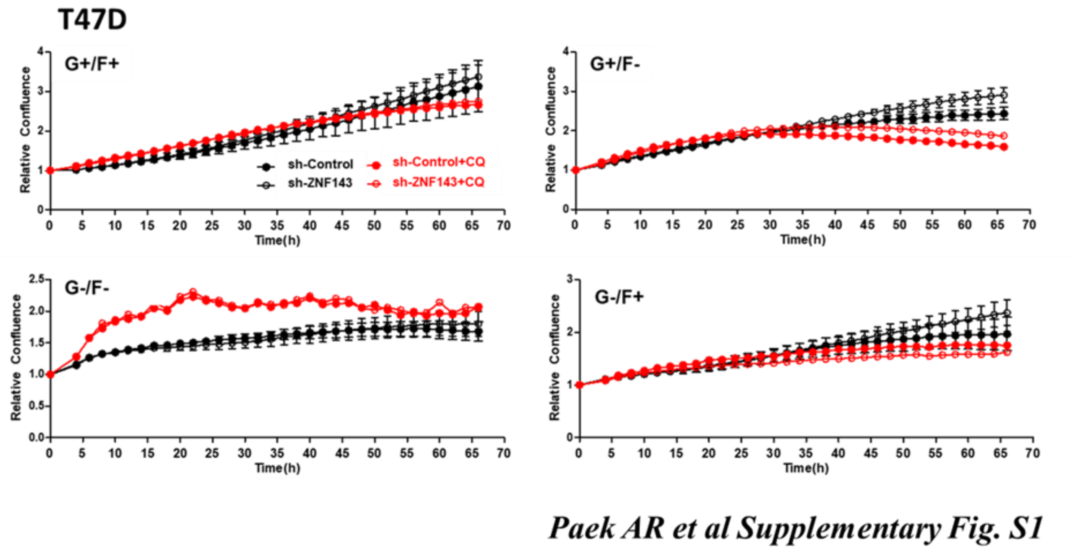

Supplmentary Figure S1. Cells were plated on 96-well plates and grown for 24 h. The cells were then maintained in four different conditions in terms of FBS and glucose, and cell survival or growth was monitored by capturing images every 2 h over 4 days. Cells were maintained in the presence of 10 μM chloroquine. Relative confluency is shown in the graphs. Data are expressed as means ± S. E. of at least three independent experiments. Statistical significance was assessed using paired Student's t-tests (*p < 0.05 and **p < 0.005). Results shown are representative of at least three independent experiments.

Supplementary Table S1. Primers for PCR

Gene	Primers (5'-3')	Reference	Product (bp)
p53-F	CCCCTCCTGGCCCCTGTCATCTTC	NM001126118.1	265bp
p53-R	GCAGCGCCTCACAACCTCCGTCAT		
ZNF143-F	CAGCATTCCATACTGCCTCA	NM003442	141bp
ZNF143-R	GAGATGGCTGTTCTCCAAGC		
NQO1-F	ATTTTGCTCCAAGCAGCCTC	NM000903.2	141bp
NQO1-R	GGTTGTCAGTTGGGATGGACT		
GAPDH-F	GGGTGTGAACCATGAGAAGT	NM33197.1	136bp
GAPDH-R	GACTGTGGTCATGAGTCCT		
PTGR1-F	CGCCAAAAAGCTCTGAAGGAC	NM001146108.1	152bp
PTGR1-R	CATGCTTTCACTATTGTCTTCCCC		
KYNU-F	GTCTCATGTAGAGGAGCGGG	NM003937.2	139bp
KYNU-R	AGGAACTGGAGCCACTCGAA		

Supplementary Table S2. Altered proteins by ZNF143 knockdown in MCF7 cells

Gene Symbol	Description
GSTP1	Glutathione S-transferase P [OS=Homo sapiens]
LDHB	L-lactate dehydrogenase B chain [OS=Homo sapiens]
S100A6	protein S100-A6 [OS=Homo sapiens]
ALDH1A1	Retinal dehydrogenase 1 [OS=Homo sapiens]
GFPT2	Glutamine--fructose-6-phosphate aminotransferase [isomerizing] 2 [OS=Homo sapiens]
AKR1C3	Aldo-keto reductase family 1 member C3 [OS=Homo sapiens]
FBXO17	F-box only protein 17 [OS=Homo sapiens]
TOP1MT	DNA topoisomerase I, mitochondrial [OS=Homo sapiens]
AKR1C1	aldo-keto reductase family 1 member C1 [OS=Homo sapiens]
BAG2	BAG family molecular chaperone regulator 2 [OS=Homo sapiens]
VN1R5	Vomeronasal type-1 receptor 5 [OS=Homo sapiens]
RAB19	Isoform 2 of Ras-related protein Rab-19 [OS=Homo sapiens]
SCIN	ADSEVERIN [OS=Homo sapiens]
C15orf26; CFAP161	Isoform 2 of Cilia- and flagella-associated protein 161 [OS=Homo sapiens]
IFNK	Interferon kappa [OS=Homo sapiens]
HBA2; HBA1	Hemoglobin subunit alpha [OS=Homo sapiens]
MAN1A1	Mannosyl-oligosaccharide 1,2-alpha-mannosidase IA [OS=Homo sapiens]
NQO1	NAD(P)H dehydrogenase [quinone] 1 [OS=Homo sapiens]
TF	Serotransferrin [OS=Homo sapiens]
ADAMTS19	A disintegrin and metalloproteinase with thrombospondin motifs 19 [OS=Homo sapiens]
KYNU	kynureninase [OS=Homo sapiens]
ALB	Serum albumin [OS=Homo sapiens]
AK1	Adenylate kinase isoenzyme 1 [OS=Homo sapiens]
LACTB	Serine beta-lactamase-like protein LACTB, mitochondrial [OS=Homo sapiens]
LXN	Latexin [OS=Homo sapiens]
ANXA1	annexin A1 [OS=Homo sapiens]
SBF1	Isoform 4 of Myotubularin-related protein 5 [OS=Homo sapiens]
TMOD2	Tropomodulin-2 [OS=Homo sapiens]
APOA1	Apolipoprotein A-I [OS=Homo sapiens]
UTP23	rRNA-processing protein UTP23 homolog [OS=Homo sapiens]
AFP	Alpha-fetoprotein [OS=Homo sapiens]
PTGR1	Prostaglandin reductase 1 [OS=Homo sapiens]
FKBP11	Peptidyl-prolyl cis-trans isomerase FKBP11 [OS=Homo sapiens]
MYO1B	Unconventional myosin-Ib [OS=Homo sapiens]
PLOD2	Isoform 2 of Procollagen-lysine,2-oxoglutarate 5-dioxygenase 2 [OS=Homo sapiens]
GNA13	Guanine nucleotide-binding protein subunit alpha-13 [OS=Homo sapiens]
CD44	CD44 antigen [OS=Homo sapiens]
SCARB1	Scavenger receptor class B member 1 [OS=Homo sapiens]
CCDC37; CFAP100	Isoform 2 of Cilia- and flagella-associated protein 100 [OS=Homo sapiens]
SFXN5	Sideroflexin-5 [OS=Homo sapiens]

MPI	mannose-6-phosphate isomerase [OS=Homo sapiens]
CKM	Creatine kinase M-type [OS=Homo sapiens]
SP3	transcription factor Sp3 [OS=Homo sapiens]
AUP1	Ancient ubiquitous protein 1 [OS=Homo sapiens]
CLDN3	Claudin-3 [OS=Homo sapiens]
RCN1	Reticulocalbin-1 [OS=Homo sapiens]
PPIF	Peptidyl-prolyl cis-trans isomerase F, mitochondrial [OS=Homo sapiens]
GNA11	guanine nucleotide-binding protein subunit alpha-11 [OS=Homo sapiens]
ATP8	ATP synthase protein 8 [OS=Homo sapiens]
FABP5	Fatty acid-binding protein, epidermal [OS=Homo sapiens]
NAT1	Arylamine N-acetyltransferase 1 [OS=Homo sapiens]
FBXO3	F-box only protein 3 [OS=Homo sapiens]
AHSG	Alpha-2-HS-glycoprotein [OS=Homo sapiens]
A2M	alpha-2-macroglobulin [OS=Homo sapiens]
CCDC175	Coiled-coil domain-containing protein 175 [OS=Homo sapiens]
MDN1	Midasin [OS=Homo sapiens]
PTRHD1	Putative peptidyl-tRNA hydrolase PTRHD1 [OS=Homo sapiens]
HEBP1	Heme-binding protein 1 [OS=Homo sapiens]
ITIH4	Inter-alpha-trypsin inhibitor heavy chain H4 [OS=Homo sapiens]
CBX1	Chromobox protein homolog 1 [OS=Homo sapiens]
DYNLT1	Dynein light chain Tctex-type 1 [OS=Homo sapiens]
LTF	Lactotransferrin [OS=Homo sapiens]
HMGN3	High mobility group nucleosome-binding domain-containing protein 3 [OS=Homo sapiens]
CYB5R1	NADH-cytochrome b5 reductase 1 [OS=Homo sapiens]
MYO5A	Isoform 3 of Unconventional myosin-Va [OS=Homo sapiens]
WDR45B; WDR45L	WD repeat domain phosphoinositide-interacting protein 3 [OS=Homo sapiens]
NCOA6	Nuclear receptor coactivator 6 [OS=Homo sapiens]
CBX2	Chromobox protein homolog 2 [OS=Homo sapiens]
C1orf198	Uncharacterized protein C1orf198 [OS=Homo sapiens]
CRELD1	Isoform 2 of Cysteine-rich with EGF-like domain protein 1 [OS=Homo sapiens]
DTL	Denticleless protein homolog [OS=Homo sapiens]
FLNB	Filamin-B [OS=Homo sapiens]
KLRG2	killer cell lectin-like receptor subfamily G member 2 [OS=Homo sapiens]
C17orf104; MEIOC	Meiosis-specific coiled-coil domain-containing protein MEIOC [OS=Homo sapiens]
NEBL	Isoform 2 of Nebulette [OS=Homo sapiens]
CTSL2; CTSV	Cathepsin L2 [OS=Homo sapiens]
ARL3	ADP-ribosylation factor-like protein 3 [OS=Homo sapiens]
PLS3	Plastin-3 [OS=Homo sapiens]
C6orf211; ARMT1	protein-glutamate O-methyltransferase [OS=Homo sapiens]
RAB31	ras-related protein rab-31 [OS=Homo sapiens]
SSFA2	Sperm-specific antigen 2 [OS=Homo sapiens]
FAM102A	Protein FAM102A [OS=Homo sapiens]
RILPL1	RILP-like protein 1 [OS=Homo sapiens]

NPDC1	Neural proliferation differentiation and control protein 1 [OS=Homo sapiens]
IDH3A	Isoform 2 of Isocitrate dehydrogenase [NAD] subunit alpha, mitochondrial [OS=Homo sapiens]
CALU	Isoform 4 of Calumenin [OS=Homo sapiens]
SERPINA3	Alpha-1-antichymotrypsin [OS=Homo sapiens]
PFN3	Profilin-3 [OS=Homo sapiens]
HOMER3	homer protein homolog 3 [OS=Homo sapiens]
LGALS1	Galectin-1 [OS=Homo sapiens]
SLC39A7	Zinc transporter SLC39A7 [OS=Homo sapiens]
TBL1XR1	F-box-like/WD repeat-containing protein TBL1XR1 [OS=Homo sapiens]
B4GALT1	Beta-1,4-galactosyltransferase 1 [OS=Homo sapiens]
ABL1	Isoform IB of Tyrosine-protein kinase ABL1 [OS=Homo sapiens]
CRIP2	Cysteine-rich protein 2 [OS=Homo sapiens]
GSKIP	GSK3-beta interaction protein [OS=Homo sapiens]
CTSD	Cathepsin D [OS=Homo sapiens]
CRAT	Carnitine O-acetyltransferase [OS=Homo sapiens]
MGLL	monoglyceride lipase [OS=Homo sapiens]
IRF2BP2	Isoform 2 of Interferon regulatory factor 2-binding protein 2 [OS=Homo sapiens]
IFT140	Intraflagellar transport protein 140 homolog [OS=Homo sapiens]
EPB41L2	band 4.1-like protein 2 [OS=Homo sapiens]
SLC25A24	Calcium-binding mitochondrial carrier protein SCaMC-1 [OS=Homo sapiens]
SDSL	serine dehydratase-like [OS=Homo sapiens]
RALGAPA2	Ral GTPase-activating protein subunit alpha-2 [OS=Homo sapiens]
BMP2K	BMP-2-inducible protein kinase [OS=Homo sapiens]
SORT1	Sortilin [OS=Homo sapiens]
TNRC6B	Trinucleotide repeat-containing gene 6B protein [OS=Homo sapiens]
LOC550643; LINC01420; NBDY	negative regulator of P-body association [OS=Homo sapiens]
GREB1	Protein GREB1 [OS=Homo sapiens]
NT5C2	Cytosolic purine 5'-nucleotidase [OS=Homo sapiens]
ZNHIT2	Zinc finger HIT domain-containing protein 2 [OS=Homo sapiens]
AGR2	Anterior gradient protein 2 homolog [OS=Homo sapiens]
CALU	Isoform 3 of Calumenin [OS=Homo sapiens]
ZRSR1	U2 small nuclear ribonucleoprotein auxiliary factor 35 kDa subunit-related protein 1 [OS=Homo sapiens]
SLITRK6	SLIT and NTRK-like protein 6 [OS=Homo sapiens]
EML2	Isoform 3 of Echinoderm microtubule-associated protein-like 2 [OS=Homo sapiens]
CHRDL1	Isoform 4 of Chordin-like protein 1 [OS=Homo sapiens]
CTSB	Cathepsin B [OS=Homo sapiens]
CLIC3	Chloride intracellular channel protein 3 [OS=Homo sapiens]
ULK1	Serine/threonine-protein kinase ULK1 [OS=Homo sapiens]
RAB6C	Ras-related protein Rab-6C [OS=Homo sapiens]
LEPREL4; P3H4	synaptonemal complex protein SC65 [OS=Homo sapiens]
BNIP3	BCL2/adenovirus E1B 19 kDa protein-interacting protein 3 [OS=Homo sapiens]

RPS6KA3	Ribosomal protein S6 kinase alpha-3 [OS=Homo sapiens]
ARMCX2	Armadillo repeat-containing X-linked protein 2 [OS=Homo sapiens]
EPS8L1	Epidermal growth factor receptor kinase substrate 8-like protein 1 [OS=Homo sapiens]
NINJ1	ninjurin-1 [OS=Homo sapiens]
ANXA6	annexin A6 [OS=Homo sapiens]
RAET1G	Retinoic acid early transcript 1G protein [OS=Homo sapiens]
NCEH1	Isoform 2 of Neutral cholesterol ester hydrolase 1 [OS=Homo sapiens]
PDXDC1; LOC102724985	Pyridoxal-dependent decarboxylase domain-containing protein 1 [OS=Homo sapiens]
TMEM120A	Transmembrane protein 120A [OS=Homo sapiens]
GLT8D1	Glycosyltransferase 8 domain-containing protein 1 [OS=Homo sapiens]
SLC3A2	Isoform 4 of 4F2 cell-surface antigen heavy chain [OS=Homo sapiens]
DAP	Death-associated protein 1 [OS=Homo sapiens]
ARFGAP3	ADP-ribosylation factor GTPase-activating protein 3 [OS=Homo sapiens]
DAAM1	Disheveled-associated activator of morphogenesis 1 [OS=Homo sapiens]
CKAP4	Cytoskeleton-associated protein 4 [OS=Homo sapiens]
ASMTL	N-acetylserotonin O-methyltransferase-like protein [OS=Homo sapiens]
LMCD1	LIM and cysteine-rich domains protein 1 [OS=Homo sapiens]
SLC7A5	large neutral amino acids transporter small subunit 1 [OS=Homo sapiens]
SCD	acyl-CoA desaturase [OS=Homo sapiens]
CHMP4C	Charged multivesicular body protein 4c [OS=Homo sapiens]
ACSL1	Long-chain-fatty-acid--CoA ligase 1 [OS=Homo sapiens]
ITGA5	Integrin alpha-5 [OS=Homo sapiens]
SLC2A1	Solute carrier family 2, facilitated glucose transporter member 1 [OS=Homo sapiens]
EVL	Isoform 1 of Ena/VASP-like protein [OS=Homo sapiens]
CBFA2T3	Protein CBFA2T3 [OS=Homo sapiens]
FAM114A1	Protein Noxp20 [OS=Homo sapiens]
ZNF185	zinc finger protein 185 [OS=Homo sapiens]
CA2	Carbonic anhydrase 2 [OS=Homo sapiens]
TIMP1	Metalloproteinase inhibitor 1 [OS=Homo sapiens]
CALML5	Calmodulin-like protein 5 [OS=Homo sapiens]
ARMCX1	Armadillo repeat-containing X-linked protein 1 [OS=Homo sapiens]
SH3BGRL	SH3 domain-binding glutamic acid-rich-like protein [OS=Homo sapiens]
HSPB8	Heat shock protein beta-8 [OS=Homo sapiens]
EMP2	Epithelial membrane protein 2 [OS=Homo sapiens]
LRRC6	protein tilB homolog [OS=Homo sapiens]
ABAT	4-aminobutyrate aminotransferase, mitochondrial [OS=Homo sapiens]
CA8	carbonic anhydrase-related protein [OS=Homo sapiens]
FOSL2	Fos-related antigen 2 [OS=Homo sapiens]
KIAA1324	UPF0577 protein KIAA1324 [OS=Homo sapiens]
ARMCX3	Armadillo repeat-containing X-linked protein 3 [OS=Homo sapiens]
ASS1	Argininosuccinate synthase [OS=Homo sapiens]
MUC1	Isoform 2 of Mucin-1 [OS=Homo sapiens]

CRABP1	Cellular retinoic acid-binding protein 1 [OS=Homo sapiens]
SUSD2	Sushi domain-containing protein 2 [OS=Homo sapiens]
GOLM1	Golgi membrane protein 1 [OS=Homo sapiens]
LYPD3	Ly6/PLAUR domain-containing protein 3 [OS=Homo sapiens]
ASPH	Aspartyl/Asparaginyl beta-hydroxylase [OS=Homo sapiens]
TFF3	Trefoil factor 3 [OS=Homo sapiens]
TFF1	Trefoil factor 1 [OS=Homo sapiens]
ADIRF; C10orf116	Adipogenesis regulatory factor [OS=Homo sapiens]
VIM	Vimentin [OS=Homo sapiens]
SLC26A2	sulfate transporter [OS=Homo sapiens]
MUC5B	Mucin-5B [OS=Homo sapiens]
